# Detection of maxillary sinusitis of endodontic origin in cone-beam CT images using deep learning algorithms

**DOI:** 10.1038/s41598-026-52147-w

**Published:** 2026-05-26

**Authors:** Omar Ayman Saleh Sherif, Nora Saif Taha, Ahmed Maged Fahmy, Huda Mohammed Ahmed Aqabat, Frank C. Setzer, Shehabeldin Saber

**Affiliations:** 1 The British University in Egypt (BUE), El Sherouk City, Egypt; 2 Cairo University, Cairo, Egypt; 3 Egyptian Maxillofacial Radiology Alliance Lab, Cairo, Egypt; 4 The British University in Egypt (BUE), El Sherouk, Cairo, Egypt; 5 University of Pennsylvania, Philadelphia, PA United States of America; 6 The British University in Egypt (BUE), El Sherouk City, Egypt

**Keywords:** Maxillary sinusitis, Odontogenic sinusitis, Maxillary sinusitis of endodontic origin, Deep learning, CBCT, Computational biology and bioinformatics, Diseases, Health care, Medical research

## Abstract

Cone-Beam Computed Tomography (CBCT) scans were retrospectively collected and examined to acquire a balanced dataset representative of Normal Maxillary Sinus (NMS), Maxillary Sinusitis of Endodontic origin (MSEO), and Maxillary Sinusitis of Non-Endodontic origin (MS-NEO) according to established criteria. Data were manually labeled, pre-processed and split into training, validation, and testing groups. A custom model workflow started by anatomic classification of input images as being coronal, sagittal, or axial, followed by segmentation of roots, lining, and sinus regions, followed by extraction of radiographic sinus features for final classification of NMS, MSEO, or MS-NEO. Model performance on testing and external datasets was performed evaluating Accuracy, Precision, Recall, F1, and DICE scores. Results showed that the anatomic classifier achieved overall Accuracy, Precision, Recall, and F1 scores of 0.99 and 0.98 on the testing and external datasets. The overall DICE scores for sagittal, coronal, and axial segmenters were 0.8, 1, and 0.9 on the testing, and 0.8, 0.9, and 0.9 on the external dataset. The features extractor achieved overall Accuracy and F1 scores of 0.9–1 for sagittal, coronal, and axial classifiers for testing and external datasets, while the Multi-View classifier exhibited excellent metrics for both datasets with potential to improve clinical diagnosis of MSEO.

**Supplementary Information:**

The online version contains supplementary material available at 10.1038/s41598-026-52147-w.

## Introduction

Maxillary sinusitis of dental origin (MSDO), also termed Odontogenic sinusitis (ODS), refers to maxillary sinusitis, with or without the extension to other paranasal sinuses secondary to either adjacent infectious dental pathology or iatrogenic injury from dental procedures^1^. Studies have shown that it accounts for 25–40% of all chronic maxillary sinusitis^2^ and 45–75% of unilateral maxillary sinus opacification on computed tomography (CT)^3^. Despite its relatively high prevalence, MSDO has received significantly little attention and has been often overlooked in sinusitis guidelines or position papers^4^.

Maxillary sinusitis of endodontic origin (MSEO) is a newer term suggested to distinguish between different odontogenic sources for sinusitis and recognize endodontic infections or apical periodontitis (AP) as an important, yet overlooked, etiologic factor due to either lack of dental symptoms or atypical radiographic presentation^5^. MSEO can be a sequalae to untreated pulpal inflammation and necrosis or can present as post-treatment disease following inadequate root canal treatment or inadequate coronal restoration of maxillary posterior teeth^6^. The integrity of the sinus mucosa may be affected by endodontic infections with or without perforation of the cortical bone of the sinus floor^7^. The clinical presentation of AP may be symptomatic or asymptomatic. Radiographically, AP has variable presentations according to the disease course, ranging from a slight widening of the periodontal ligament space to well-defined radiolucent lesions around a tooth apex. Subtle radiographic changes are not always detectable on medical CT images which may be more familiar to radiologists and physicians^8^ and can be also discounted in two-dimensional (2D) radiographs due to the superimposition of anatomical structures^9^. Minor periapical changes may only be evident using small field of view cone beam computed tomography (CBCT) examination that allows visualizing both bone and soft tissue changes in multiple plans with a thin slice thickness^10^.

Cumulative evidence has demonstrated that CBCT imaging enables a better understanding and classification of the spatial relationships between root apices/periapical lesions and the maxillary sinus^11,12^.Thus, CBCT may also aid in the diagnosis and treatment planning for the cure of ODS. Unfortunately, radiographic examinations, including the evaluation of dental CBCT images, suffer from limited inter- and intra-examiner reliability, which further depends on the examiner’s experience^13^. Therefore, a clear need exists for automated, Artificial Intelligence (AI) supported assistance for dental radiographic interpretation that may enhance diagnostic precision, provide an unbiased secondary review, and help streamlining clinical workflows for the benefit of patients and practitioners^14^.

Indeed, AI is emerging as a promising approach to modernise traditional aspects of dentistry with reduced interpretation bias^15^. Deep learning (DL), a major subset of AI has been consistently implemented for biomedical image analysis. It consists of several computational layers of neural networks that can automatically detect, segment, and classify radiographic features^16^. To date, diagnostic and therapeutic AI tools have been developed to support the clinical decision-making process and have shown promising results in different medical disciplines^17^.

Previous studies attempted maxillary sinus segmentation and detection of sinus pathology using different AI algorithms^18^. However, research specific to the application of AI to MSEO-related features is limited, highlighting a critical gap in the knowledge regarding the leverage of AI to assist in diagnosing and managing this condition. An AI-driven approach may bridge this gap and significantly improve early detection and treatment planning to improve operator and patient-based outcomes. Therefore, the aim of this study was to develop AI-supported, automated models based on state-of-the-art DL algorithms to segment sinus regions and to classify sinus conditions using single or multiple CBCT images as input data. The null hypothesis was that the proposed multi-planar DL pipeline would not demonstrate significant differences in diagnostic accuracy for the classification of maxillary sinus conditions (NMS, MSEO,MS-NEO) when compared to the reference standard established by experienced clinical examiners.

## Methods

### Study approval

This prospective study was approved by the research ethics committee of the Faculty of Dentistry at The British University in Egypt (Approval number: 24–075 on 10/12/2024). The investigation strictly adhered to the World Medical Association Declaration of Helsinki on medical research and followed the CLAIM guidelines^19^. Informed consent for data usage was obtained from all subjects.

### Data acquisition

A total of 70 small field of view (5 × 5 mm) CBCT scans were retrospectively collected from two specialized endodontic clinics during the period 1/1/2022 to 31/3/2025. No CBCT images were taken specifically for this study. The scans were acquired using different acquisition parameters for a variety of clinical reasons not related to this study, which ensured heterogeneous sampling (Supplementary Table 1). To ensure dataset quality and improve model accuracy, scans were excluded if they had motion artifacts, high scatter/metal artifacts, low contrast that fail to show the necessary anatomical details or pathological/anatomical alterations that could disrupt the segmentation and classification algorithms.

### Diagnostic criteria

The CBCT scans were manually de-identified using the proprietary software (Planmeca Romexis version 6; Planmeca Oy, Helsinki, Finland) and exported as Digital Imaging and Communications in Medicine files (DICOM). Prior to image selection and export, all CBCT volumes were realigned according to the long axis of the tooth of interest using the multiplanar reconstruction tools integrated within the Planmeca Romexis software, ensuring consistent anatomical orientation across all cases and minimizing geometric distortion during subsequent analysis.

All CBCT volumes were assessed dynamically, with examiners actively scrolling through the entire volume across the axial, coronal, and sagittal planes each providing distinct and complementary anatomical perspectives of the maxillary sinus and surrounding dental structures. Representative 2D reconstructed slices were systematically selected from each plane according to the following predefined diagnostic criteria: Normal maxillary sinus (NMS): the maxillary sinus exhibits no mucosal thickening on the CBCT images or demonstrates a uniform mucosal thickening ≤ 2 mm. Maxillary sinusitis of endodontic origin (MSEO): presence of mucosal thickening, a halo or dome-shaped localized soft-tissue expansion in the floor of the sinus directly adjacent to an infected root apex^20^, Maxillary sinusitis of non-endodontic origin (MS-NEO): presence of generalized, symmetrical mucosal thickening or a soft-tissue density mass within the sinus, not limited to any tooth. Mucosal thickening was measured at the point of maximum thickness from the sinus floor using the measurement tool provided in the Planmeca Romexis software.

The selected slices were exported as discrete static 2D images for input into the DL pipeline. Following systematic dynamic review of all CBCT volumes, a balanced dataset comprising 5,152 2D reconstructed CBCT images was compiled, representing the three diagnostic categories across the axial, coronal, and sagittal planes.

Radiographic interpretation and diagnostic labeling were performed independently by a panel of three experienced clinicians: two endodontists (SS, HA) and one oral radiologist (NS) with more than 20 years of clinical and academic experience. The examiners were first calibrated by examining five cases. Images were viewed on an 18.5-inch HD LED monitor (Lenovo, Shenzhen, China) with resolution of 1366 × 768 under controlled and consistent environmental lighting conditions. Zoom, brightness and contrast tools were available for use. The examiners took a break of ten minutes after scoring one case. Any disagreement was resolved through discussion between the three examiners to reach a consensus. The radiographs were re-scored at a 1-month interval to assess the intra- and inter-observer agreements using kappa values which were 0.95 and 0.96, respectively. Consensus was reached in all cases.

### Data annotation

**Fig. 1:**
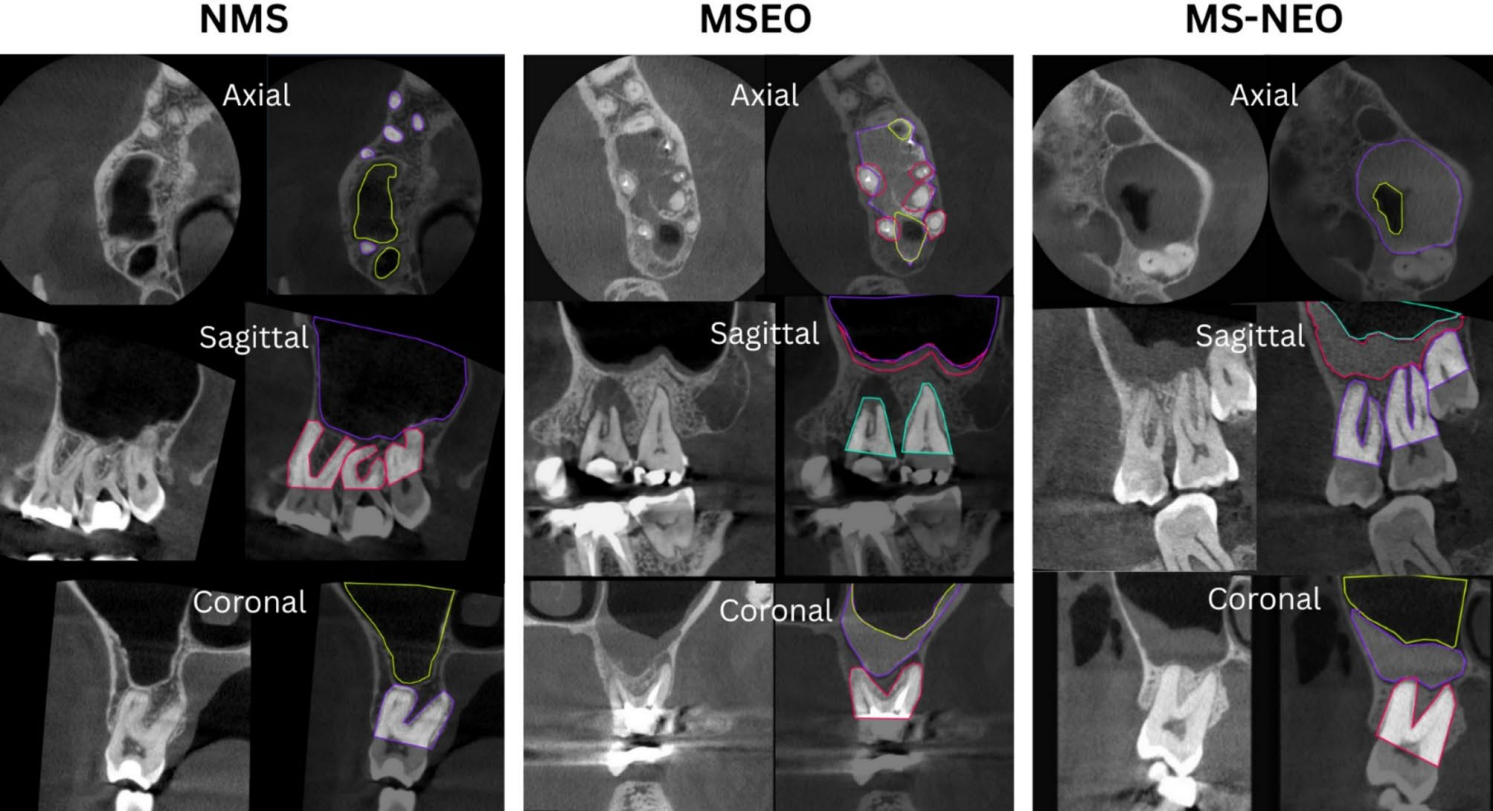
Roboflow’s polygon annotation tool used to manually annotate the sinus, its lining, as well as the adjacent roots on the three orthogonal planes, axial, sagittal and coronal for the three categories, normal maxillary sinus (NMS), maxillary sinusitis of endodontic origin (MSEO), and maxillary sinusitis of non0endodontic origin (MS-NEO).

Data were uploaded for manual labelling using Roboflow (Version 1.0) [Software]. Available from https://roboflow.com. computer vision. [Preprint]. https://roboflow.com (Accessed: January 1, 2025)] which is an online platform for computer vision tasks that can process a dataset of images and their labels in different domains^21^. The polygon annotation tool was used to manually annotate the sinus, its lining, as well as the adjacent roots as shown in Fig. 1.

### Data pre-processing

**Fig. 2:**
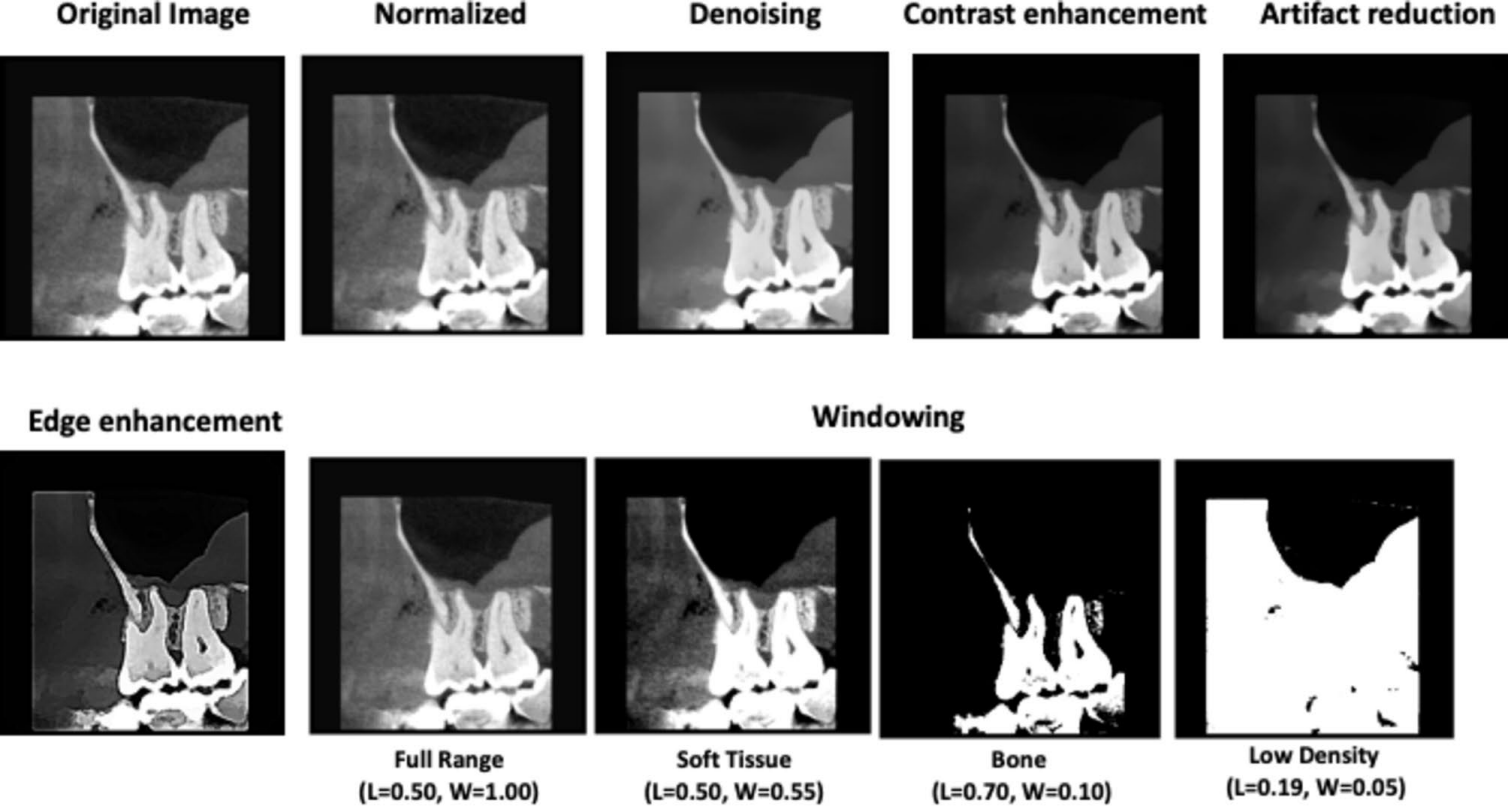
Digital image pre-processing stages of grey-scale radiographs.

**Fig. 3:**
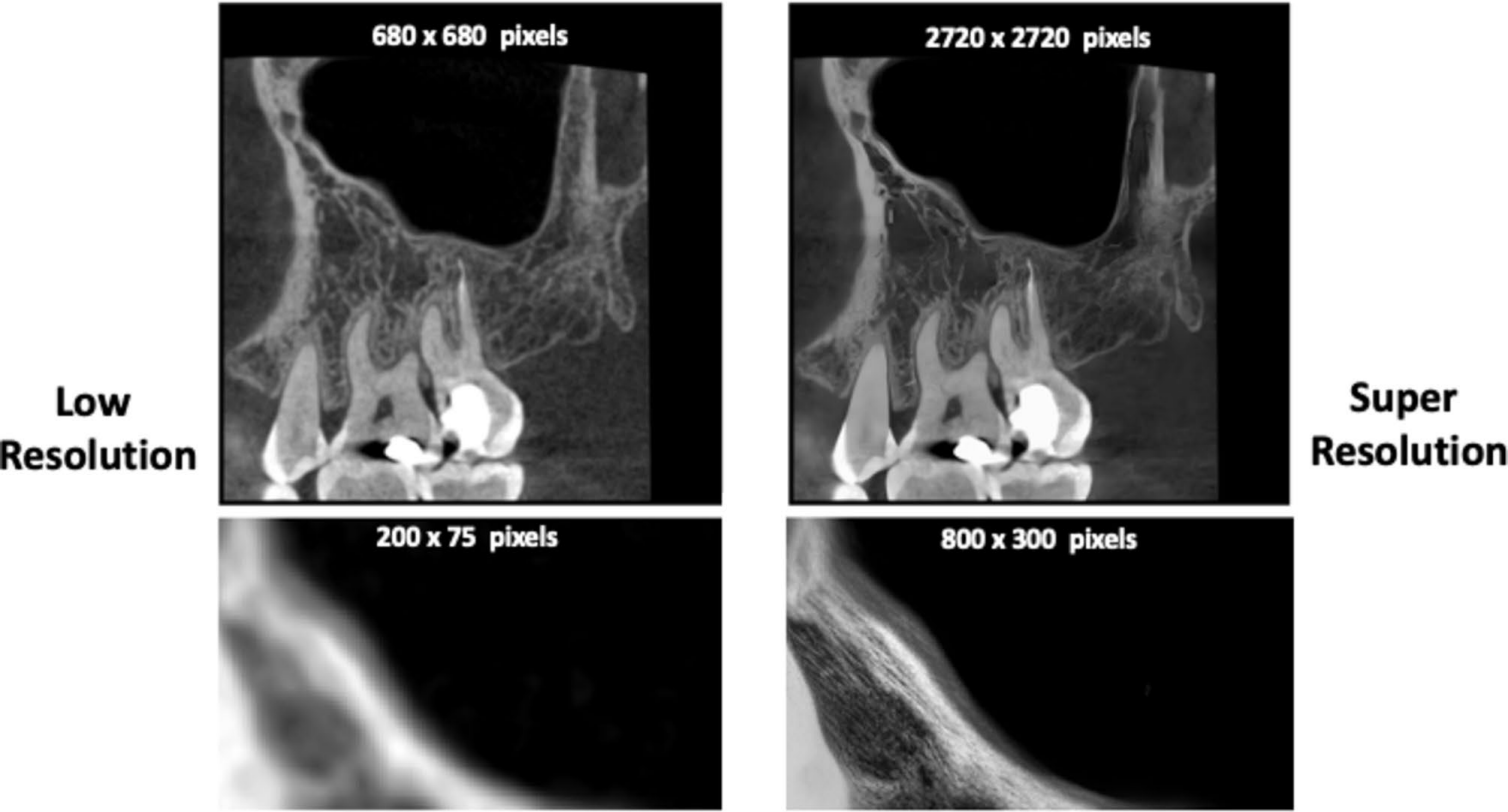
Super-resolution images generated by the super-resolution diffusion model.

To prevent data leakage, the dataset was split in a patient-wise manner into training (70%), validation (15%), and testing (15%) subsets. No patient contributed images to more than one subset. The complete patient-level allocation, including patient numbers, subset assignments, and image counts is detailed in (Supplementary Table 2). Before feeding the annotated images into the DL model pipeline, all grey-scale radiographs were digitally pre-processed through standardization of image size to (640 × 640) pixels, denoising, contrast enhancement, artifact reduction, edge enhancement and windowing (Fig. 2). Subsequently, a super-resolution diffusion model generated a downscaled (4x) version of the training dataset through supervised learning to generate super resolution images (Fig. 3) with less computational requirements for training.

Furthermore, geometric and photometric image augmentation techniques were applied exclusively to the training set to address discrepancies and improve dataset robustness, including Rotate, Shift Scale Rotate, Horizontal Flip, Random Brightness Contrast, Gauss Noise, Gaussian Blur, CLAHE, Random Gamma, and Grid Dropout as augmentation techniques. The generative approach employed a custom diffusion model with an attention block in the U-net architecture trained to generate images of the three classes. Collectively, this resulted in the addition of 1000 images per each class (NMS,MSEO, and MS-NEO).

### DL model workflow

**Fig. 4:**
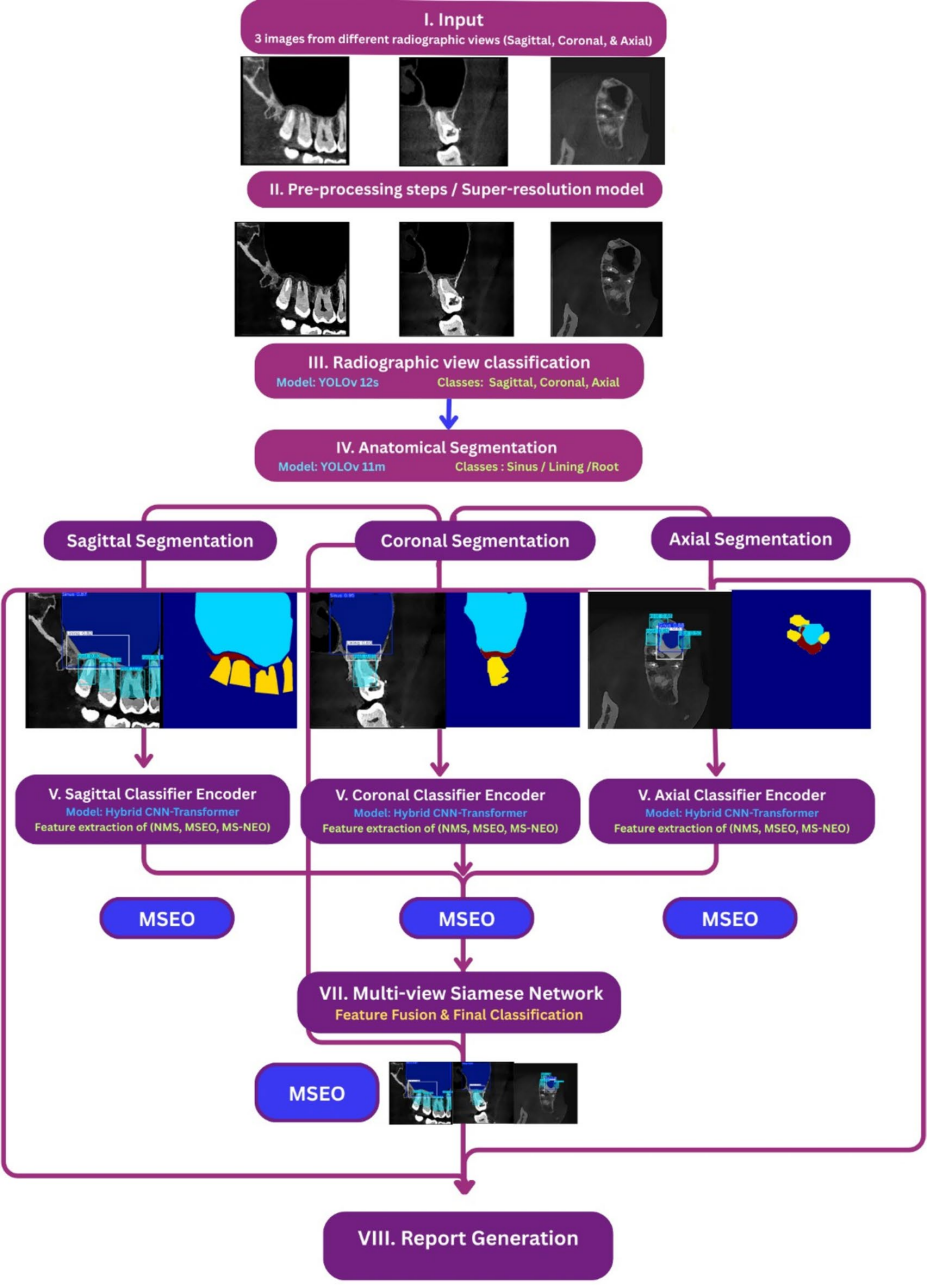
Deep learning model workflow.

The proposed diagnostic workflow (Fig. 4) received three simultaneously acquired 2D reconstructed CBCT images, representing the axial, coronal, and sagittal planes as a unified multi-planar input for each clinical case. Image were independently processed by a YOLOv12s-based radiographic view classifier, which assigned each image to its corresponding anatomical plane prior to downstream processing. The classified images were subsequently passed to a YOLOv11m segmentation model, which delineated three anatomical regions of interest within each image: the tooth roots, the sinus mucosal lining, and the sinus cavity. The resulting masked images from each plane were then forwarded to their respective plane-specific encoder, implemented as a custom Hybrid CNN-Transformer architecture, for the extraction of high-dimensional feature representations indicative of the underlying sinus condition. The extracted feature vectors from all three planes were subsequently integrated within a custom Multi-View Siamese Network (MVSN), which fused the spatial information across all three orthogonal views to generate a single unified sinus classification output (NMS, MSEO, or MS-NEO). Finally, the classification output, together with the corresponding segmented images, was passed to a multimodal model, (which synthesized both the visual and categorical information) to generate a structured, comprehensive diagnostic report.

## Custom models architecture

### The hybrid CNN-transformer (Supplementary Fig. 1)

#### CNN backbone

The CNN backbone consisted of eight Residual CNN Blocks based on the ResNet architecture that used residual connections to extract hierarchical features of the input data. The shallower blocks identified simple shapes and textures, such as bone structures, soft tissue boundaries, whereas deeper blocks identify more complicated patterns, such as mucosal thickening, opacifications, or the presence of periapical lesions. Each block had a channel attention, which allowed for a dynamic representation of learned features. The architecture used Batch Normalization and Dropout to help the model be less prone to variations in image quality, patient anatomy, and possible noise, resulting in improved generalization.

#### Transformer

A stack of six Transformer Blocks ran the input sequence (the flattened and embedded CNN features) through two primary sub-layers in series, a Multi-Head Self-Attention (MHSA) mechanism, and a position-wise Feed-Forward Network (FFN), with residual connections and layer normalization around each. This architecture enabled the model to capture intricate dependencies in the sequence whilst having stable training.

### The multi view siamese network (MVSN)

The advanced MVSN architecture (Supplementary Figure 2) designed for tasks that involve comparing multiple views or representations of the same object was employed for classification improvement.

### Model hyperparameters

For all models, a Bayesian Optimization technique was used in a high search space to identify the optimal combination of hyperparameters (Supplementary Table 3).

### External validation

Following the evaluation of the developed models’ performance on the testing dataset, a new set of 738 2D CBCT images (coronal, sagittal, axial images) was obtained to conduct an external validation of the trained model.

### Evaluation metrics

A comprehensive set of evaluation metrics was used to assess the performance of the present models. For classification, accuracy, precision, recall, and F1-score were used to provide a robust evaluation of model performance across various aspects of predictive accuracy and error types. For the segmentation tasks, the Dice score was employed to measure the overlap between predicted and ground truth segmentations, alongside object detection and instance segmentation metrics such as Box Precision (Box P), Box Recall (Box R), Box mAP50, and Box mAP50-95 for bounding box evaluation, and Mask Precision (Mask P), Mask Recall (Mask R), Mask mAP50, and Mask mAP50-95 for mask-based segmentation, ensuring a thorough assessment of both localization and segmentation quality.

### Computation resources

NVIDIA GeForce RTX 4060 Ti 8 GPU (Nvidia, USA).

## Results

**Fig. 5:**
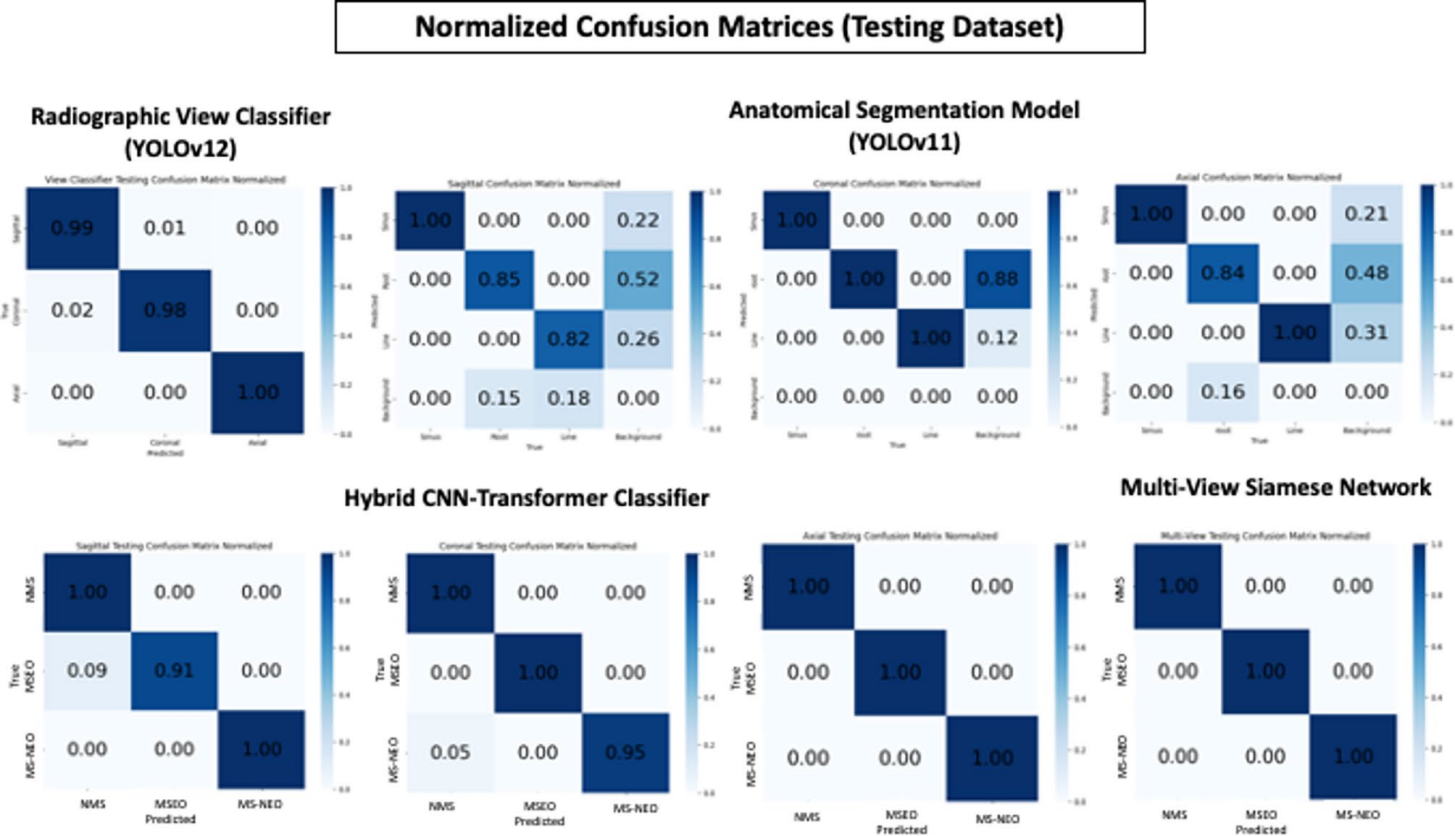
Normalized confusion matrices showing the performance of different models on the testing dataset.

**Fig. 6:**
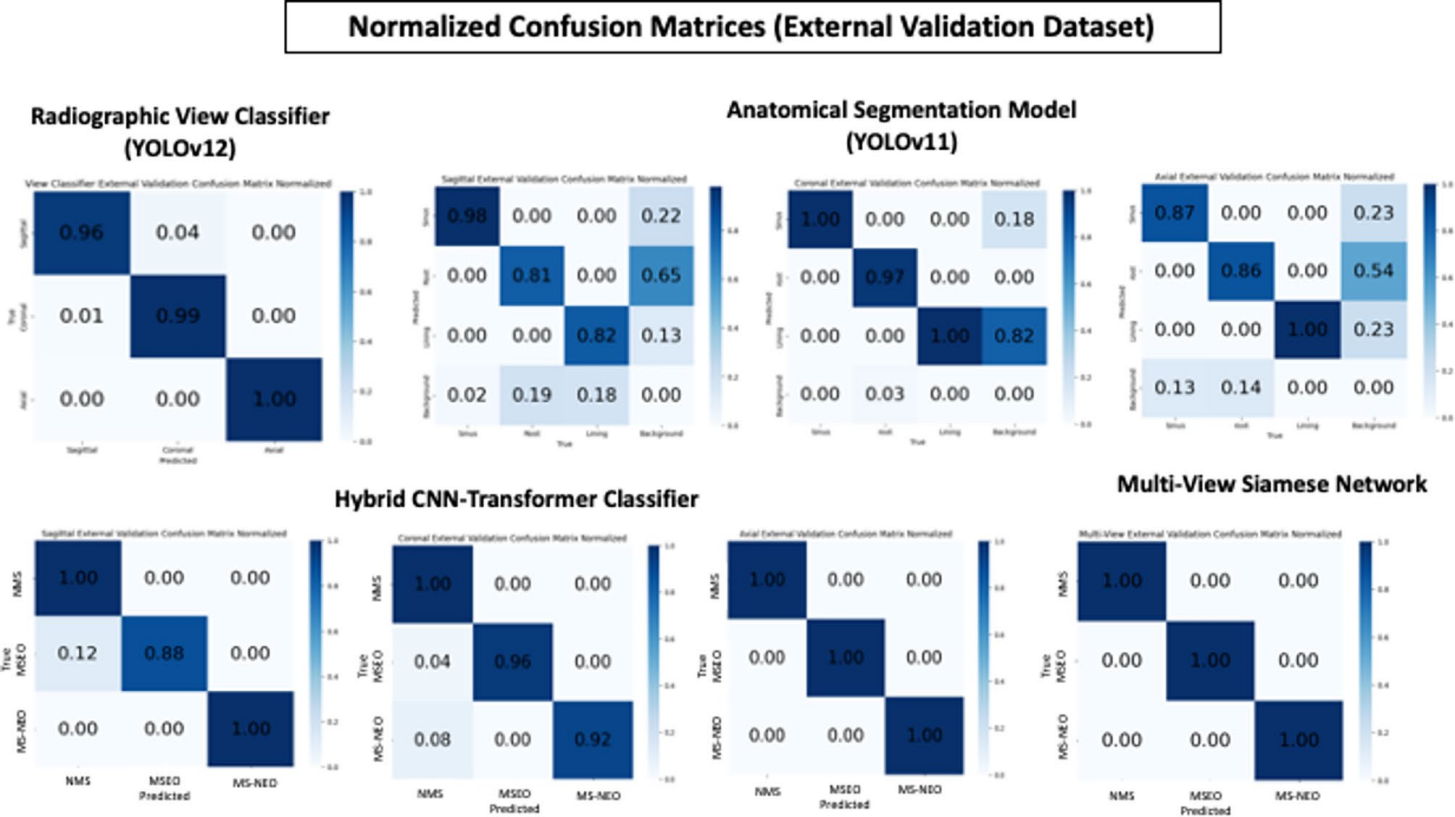
Normalized confusion matrices showing the performance of different models on the external validation dataset.

**Fig. 7:**
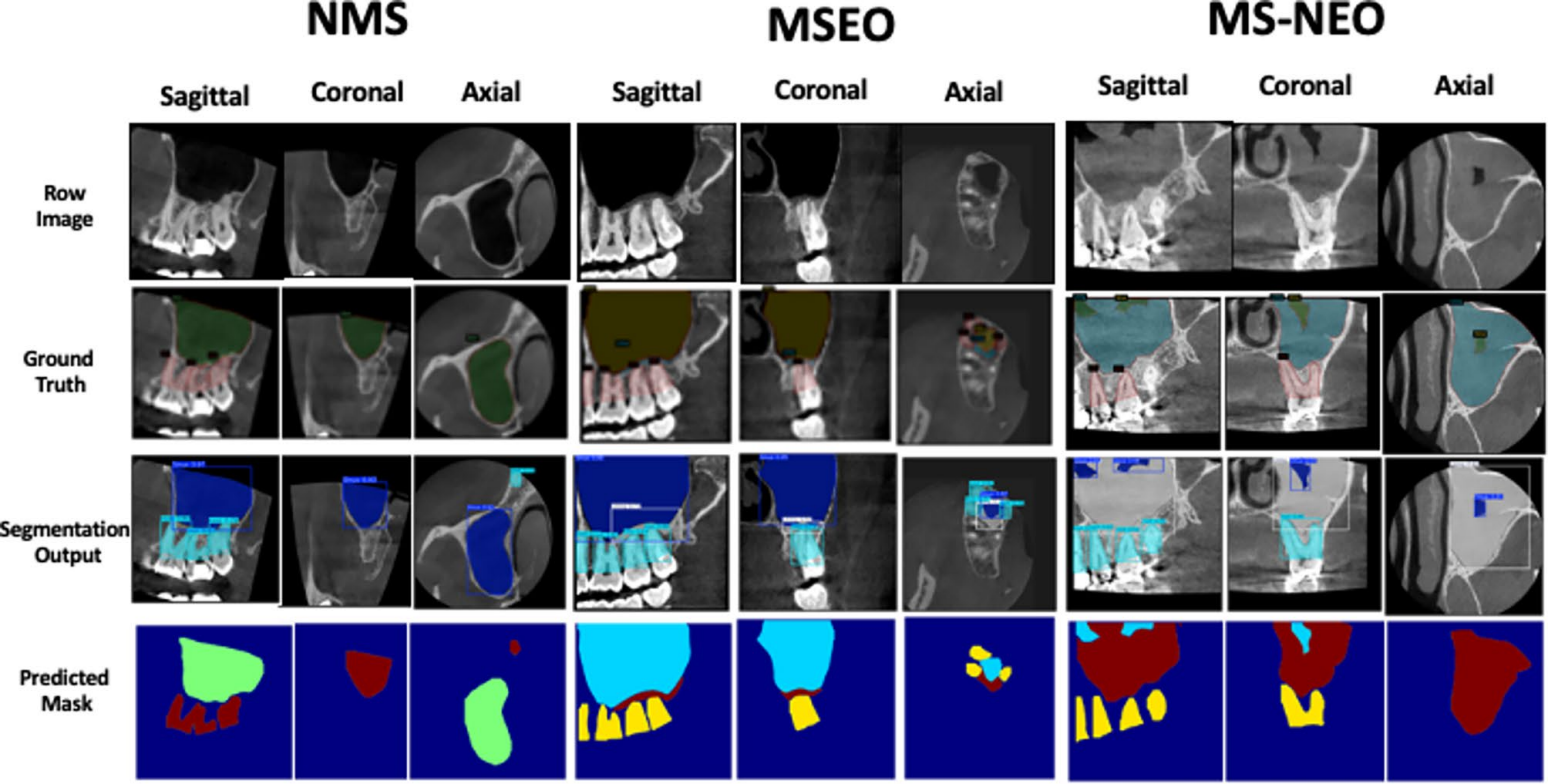
Representative images for all models’ predictions across all views and all classes of the testing dataset.

The performance metrics and confusion matrices of all trained models evaluated on the testing and external validation datasets are presented in Tables 1–4 and Figs. 5 and 6, with representative prediction outputs across all views and diagnostic classes illustrated in Fig. 7.

**Table 1:** Performance metrics of the anatomical segmentation model (YOLOv11m) on the testing dataset.

class	Box (P		R	mAP50	mAP50-95)	Mask (P	R	mAP50	mAP50-95)	DICE score
Sagittal view segmenter										
Sinus	0.94		0.995	0.993	0.934	0.941	0.99	0.993	0.909	1
Root	0.928		0.785	0.993	0.535	0.921	0.761	0.916	0.439	0.837
Lining	0.968		0.775	0.826	0.552	0.968	0.761	0.822	0.439	0.832
Coronal view segmenter										
All	0.922		0.992	0.983	0.755	0.922	0.992	0.983	0.764	1
Sinus	0.997		1	0.995	0.894	0.997	1	0.995	0.898	1
Root	0.879		0.976	0.97	0.734	0.879	0.976	0.97	0.655	1
Lining	0.89		1	0.983	0.636	0.89	1	0.983	0.739	1
Axial view segmenter										
All	0.74		0.947	0.939	0.678	0.74	0.947	0.939	0.678	0.946
Sinus	0.904		1	0.994	0.715	0.904	1	0.994	0.734	1
Root	0.737		0.841	0.829	0.453	0.737	0.841	0.829	0.495	0.913
Lining	0.578		1	0.995	0.866	0.578	1	0.995	0.806	0.925

Box P = Box Precision, Box R = Box Recall, mAP50 = mean Average Precision at Intersection over Union (IoU) threshold of 0.50, mAP50-95 = mean Average Precision averaged over IoU thresholds from 0.50 to 0.95 in increments of 0.05, DICE score = Dice Similarity Coefficient, measuring the overlap between predicted and ground truth segmentations.

**Table 2:** Performance metrics of the Hybrid CNN-transformer model on the testing dataset

	Accuracy	Precision	Recall	F1 Score
Sagittal view classifier				
NMS	-	0.9174	1	0.9569
MSEO	-	1	0.91	0.9529
MS-NEO	-	1	1	1
Overall	0.97	0.9725	0.97	0.9699
Coronal view classifier				
NMS	-	0.9524	1	0.9756
MSEO	-	1	1	1
MS-NEO	-	1	0.95	0.9744
Overall	0.9833	0.9841	0.9833	0.9833
Axial view classifier				
NMS	-	1	1	1
MSEO	-	1	1	1
MS-NEO	-	1	1	1
Overall	1	1	1	1

**Table 3:** Performance metrics of the anatomical segmentation model (YOLOv11m) on the external validation dataset.

class	Box P	Box R	Box mAP50	Box mAP50-95)	Mask P	MaskR	Mask mAP50	Mask mAP50-95)	DICE score
Sagittal view segmenter									
All	0.928	0.849	0.907	0.652	0.891	0.839	0.874	0.586	0.8701
Sinus	0.947	0.981	0.993	0.932	0.938	0.981	0.994	0.982	0.907
Root	0.886	0.758	0.895	0.524	0.891	0.795	0.894	0.448	0.895
Lining	0.952	0.888	0.833	0.5	0.845	0.742	0.734	0.41	0.807
Coronal view segmenter									
All	0.964	0.96	0.982	0.973	0.962	0.958	0.981	0.745	0.994
Sinus	0.998	1	0.995	0.924	0.998	1	0.995	0.901	1
Root	0.922	0.881	0.957	0.727	0.914	0.873	0.954	0.665	0.984
Lining	0.973	1	0.995	0.541	0.973	1	0.995	0.67	1
Axial view segmenter									
All	0.925	0.856	0.949	0.635	0.937	0.854	0.951	0.606	0.912
Sinus	0.989	0.85	0.959	0.611	1	0.858	0.974	0.624	0.930
Root	0.863	0.718	0.893	0.401	0.886	0.705	0.885	0.417	0.924
Lining	0.924	1	0.995	0.892	0.925	1	0.995	0.778	0.881

Box P = Box Precision, Box R = Box Recall, mAP50 = mean Average Precision at Intersection over Union (IoU) threshold of 0.50, mAP50-95 = mean Average Precision averaged over IoU thresholds from 0.50 to 0.95 in increments of 0.05, Mask P = Mask Precision, Mask R = Mask Recall, DICE score = Dice Similarity Coefficient, measuring the overlap between predicted and ground truth segmentations.

**Table 4:** Performance metrics of the Hybrid CNN-Transformer model on the testing dataset.

	Accuracy	Precision	Recall	F1 score
Sagittal view classifier				
NMS	-	0.8929	1	0.9434
MSEO	-	1	0.88	0.9362
MS-NEO	-	1	1	1
Overall	0.96	0.9643	0.96	0.9599
Coronal view classifier				
NMS	-	0.8929	1	0.9434
MSEO	-	1	0.96	0.9796
MS-NEO	-	1	0.92	0.9583
Overall	0.96	0.9643	0.96	0.9604
Axial view classifier				
NMS	1	1	1	1
MSEO	1	1	1	1
MS-NEO	1	1	1	1
Overall	1	1	1	1

### Radiographic view classifier (YOLOv12s)

The radiographic view classifier demonstrated excellent discriminative performance, achieving overall accuracy, precision, recall, and F1 scores of 0.99 and 0.98 on the testing and external validation datasets, respectively.

These results confirm the model’s capacity to reliably identify the anatomical plane of each input image (axial, coronal, or sagittal) with a high degree of certainty, thereby ensuring the integrity of all subsequent processing stages within the pipeline.

### Segmentation model (YOLOv11m)

The segmentation model yielded strong performance across all three plane-specific segmenters. On the testing dataset (Table 1); the overall DICE scores were 0.89, 1.00, and 0.94 for the sagittal, coronal, and axial segmenters, respectively. While, on the external validation dataset (Table 3); corresponding DICE scores were 0.87, 0.99, and 0.91, indicating consistent and robust segmentation performance across both datasets. These findings indicate that the model reliably delineated the three anatomical regions of interest — the tooth roots, sinus mucosal lining, and sinus cavity — across both datasets and all orthogonal planes .

### Plane-specific classifiers (hybrid CNN-transformer)

The plane-specific Hybrid CNN-Transformer classifiers demonstrated strong individual diagnostic performance, with notable variation across anatomical planes (Tables 2, 4). The sagittal classifier achieved an overall accuracy and F1 score of (0.97, 0.96) on the testing dataset, and (0.96, 0.95) on the external validation dataset. The coronal classifier attained an overall accuracy and F1 scores of 0.9 on both datasets. The axial classifier demonstrated the strongest individual performance, achieving perfect overall accuracy and F1 scores of 1.00 on both the testing and external validation datasets.

### Multi-view Siamese network (MVSN)

The MVSN achieved perfect classification performance, attaining accuracy, precision, recall, and F1 scores of 1.00 across all three diagnostic classes, NMS, MSEO, and MS-NEO, on both the testing and external validation datasets, as evidenced by the perfect diagonal of 1.00 values in the corresponding confusion matrices (Figs. 5,6). The perfect classification performance achieved by the MVSN represented a remarkable improvement over any individual plane-specific classifier, underscoring the clinical and diagnostic value of orthogonal multi-planar feature fusion in automated sinus condition assessment.

According to these findings, the null hypothesis cannot be rejected as the proposed pipeline achieved perfect agreement with the ground truth diagnoses established through consensus by a panel of experienced clinical examiners, whose diagnostic reliability was confirmed by near-perfect intra- and inter-observer kappa coefficients of 0.95 and 0.96, respectively .

## Discussion

Dental practitioners may lack the training and expertise to accurately identify sinusitis on biomedical images and determine its etiology. Moreover, the accurate analysis of complex anatomical structures, such as the maxillary sinus and its surroundings, requires the processing of images in multi-dimensions. This is time-consuming, laborious, and eventually highly dependent on experts’ knowledge and experience^22^. Therefore, there is a need for automated, AI-supported image analysis for the detection and classification of sinusitis. The present study sought to develop an automated DL model to classify the radiographic features associated with MSEO based on input CBCT data, which should be combined with thorough case history and clinical examination to reach a definitive diagnosis.

Generally, three-dimensional imaging modalities are preferred to study maxillary sinus anatomy and pathology^12^. Although Computed Tomography (CT) is widely used in medical offices and offers high quality images with good spatial resolution, CBCT offers imaging with higher resolution, excellent detail of bony structures, significantly lower radiation dose, shorter scanning time, and lower cost^23^. Therefore, dental CBCT may be generally more adequate for automated image analysis regarding the maxillary sinus. Moreover, it is the most common three-dimensional (3D) imaging technique used by general dental practitioners, dental specialists, and oral surgeons^24^. Hence, we chose CBCT as the 3D imaging modality to acquire our dataset. To ensure the robustness and generalizability of deep learning (DL) models, we sought to use high-quality annotated data that is as heterogeneous as possible^25^.

Overall, the trained models demonstrated excellent predictive performance on both the training and validation datasets. This is evident from the confusion matrices, which show high true positive and true negative rates while minimizing false positives and false negatives. Such performance can be attributed to several factors. First, the distinctive radiographic signatures of the three classes which exhibit fundamentally different spatial patterns when viewed across all three anatomical planes simultaneously. Second, the high-quality ground truth labeling established by consensus among domain experts with more than 20 years of clinical experience and achieving consensus in all cases. This rigorous labeling process reduced noise in the training labels, which is a known factor in enabling higher model performance. Third, the comprehensive image pre-processing pipeline and the custom super-resolution diffusion model applied prior to model training. Raw CBCT images inherently suffer from noise, low soft-tissue contrast, and artifacts from metallic restorations, all of which obscure diagnostically relevant features and hinder automated classification. Our multi-stage pre-processing pipeline systematically addressed each of these limitations: denoising removed random noise that could mask subtle mucosal changes; contrast enhancement amplified the visibility of soft-tissue boundaries and mucosal thickening relative to surrounding structures; the threshold-based artifact reduction method identified and replaced high-intensity pixels caused by metallic objects with median values from surrounding non-artifact regions, eliminating streak artifacts that would otherwise confuse the classifier; and edge enhancement using Unsharp Masking, Sobel, and Laplacian operators sharpened the boundaries of the sinus wall, mucosal lining, and root outlines, making these diagnostically critical structures more prominent for feature extraction. Additionally, windowing with optimized parameters for soft tissue (L = 0.50, W = 0.55) and bone (L = 0.70, W = 0.10) selectively enhanced the contrast of specific tissue density ranges most relevant to the diagnostic task. Beyond pre-processing, the custom super-resolution diffusion model played a substantial role in enriching the discriminative features available to the classifier. By upscaling training images by a factor of 4 ×, the super-resolution model recovered and enhanced fine-grained anatomical details such as the precise morphology of the mucosal lining, the subtle transition zone between healthy and thickened mucosa, and the detailed periapical anatomy at the root apex-sinus floor interface that are critical for distinguishing MSEO from NMS and MS-NEO but are often indiscernible at native CBCT resolution. This enrichment of diagnostically relevant features effectively increased the separability of the three classes in the feature space, enabling the downstream classifiers to make more confident and accurate decisions. The combination of systematic noise and artifact suppression, targeted feature enhancement, and super-resolution upscaling collectively transformed the raw CBCT data into a highly optimized input representation in which the distinguishing radiographic signatures of the three sinus conditions were maximally expressed, thereby directly contributing to the strong classification performance observed across all models.

Finally, the architectural features of each algorithm used throughout the model workflow. For the radiographic view classifier, the advanced modules and attention mechanisms integrated into the YOLOv12 architecture significantly enhanced its detection and classification^26^. Similarly, the intrinsic features of YOLOv11, particularly the C3k2 block, SPPF for multi-scale features, and C2PSA, improved its spatial attention and segmentation capabilities^27^. The custom Hybrid CNN-Transformer capitalized on the strengths of both CNNs and Transformers to extract hierarchical features from image data. While channel attention blocks in CNNs excel at capturing local patterns, they are limited by a restricted receptive field. In contrast, Transformers, through their self-attention mechanisms, allow the model to assign weights to the importance of different segments of the input sequence, thereby enhancing the learning of global connections^28^. As for the MVSN, the proposed architecture facilitates feature extraction from multiple images from three independent classifiers, one for each anatomical plane (sagittal, coronal, and axial). The individual Hybrid CNN-Transformer classifiers already achieved high but imperfect accuracy (0.96–1.0 across views and datasets). When features from all three planes are fused through the cross-view attention blocks and Multi-Layer Perceptron of the MVSN, the complementary information from different anatomical perspectives compensates for the errors of individual classifiers, thereby increasing the model’s capacity to learn complex patterns^29^. The effectiveness of these architectural choices is reflected in the analysis of the confusion matrices across various models. On several occasions, segmenters and classifiers achieved matrices with either no prediction errors or only minor errors.

The confusion matrix of the MVSN displayed a perfect diagonal of 1.00 values across all classes. In practical applications, such an ideal confusion matrix is exceedingly rare and may imply model overfitting or that the dataset is exceptionally simple or easily separable. Nonetheless, in the current study, multiple comprehensive strategies were employed to avoid anti-overfitting.

This included geometric and photometric data augmentation techniques, exclusively applied to the training set to increase data diversity and force the models to generalize beyond the specific training such as: generative data augmentation through a custom diffusion model generating 1,000 additional synthetic images per class further expanding the training distribution; several regularization techniques (Stochastic depth regularization, batch normalization, DropConnect, and dropout) that were all incorporated into the model architectures to reduce co-adaptation of neurons and prevent memorization of training patterns; the application of gradient clipping to stabilize training and prevent sharp weight updates that could lead to overfitting on specific examples; label smoothing with CrossEntropyLoss in the MVSN to prevent the model from becoming overconfident in its predictions thereby promoting better generalization; weight decay (ranging from 1e-4 to 5e-4 across different models) applied through the AdamW optimizer to penalize large weights and discourage model complexity; mixed precision training with GradScaler, which not only accelerated training but also introduces beneficial numerical noise that acted as an implicit regularizer; early stopping. implemented with patience values of 15–100 epochs, depending on the model to halt training when validation performance plateaued thereby preventing the model from continuing to optimize on training data after useful learning had ceased; Stochastic Weight Averaging (SWA), initiated at epoch 30 in the Hybrid CNN-Transformer with a frequency of 5 epochs, which averaged model weights across multiple training epochs and had been shown to improve generalization by finding broader optima in the loss landscape; Learning rate scheduling using ReduceLROnPlateau (with factor 0.3–0.5 and patience 5–15 epochs) applied to dynamically reduce the learning rate when validation loss stopped improving, and preventing the model from oscillating around sharp minima that generalize poorly.

Furthermore, data splitting was stratified by patients to prevent data leakage, which may be one of the most common sources of artificially inflated performances in DL studies. In our study, we implemented strict patient-wise data splitting to ensure that all images derived from a single CBCT scan (and thus a single patient) were assigned exclusively to either the training, validation, or testing subsets. No patient contributed images to more than one subset under any circumstance. This approach is fully detailed in Supplementary Table 2, which explicitly lists every patient number (PN) and the subset to which they were assigned, along with the exact image count per patient. Furthermore, data augmentation and synthetic image generation were applied exclusively to the training set. No augmentation was performed on the validation or testing sets, thereby ensuring that no artificially generated variants of training images could appear in the evaluation datasets.

Previous studies attempted maxillary sinus segmentation and detection of sinus pathology using different algorithms; Jung et al^30^. segmented the air area and the lesion area in the maxillary sinus and obtained a dice similarity coefficient (DSCs) of 0.93 and 0.76, respectively. However, the algorithm proposed was not designed to differentiate between Mucosal Thickening (MT) and Mucus retention cysts (MRC). Choi et al.^31^ proposed a model to segment hazy and clear maxillary sinuses using a U-Net and achieved a DSC of 0.9. However, the CBCT images were acquired from a single center. Altun et al.^32^ used a YOLOv5 architecture to automatically segment the maxillary sinus and identify MRC, MT, total, and partial opacifications, and healthy sinuses. Recall, precision, and F1 score values for total maxillary sinus segmentation were 1, 0.985, and 0.992, respectively; 1, 0.931, and 0.964 for healthy maxillary sinus segmentation; 0.858, 0.923, and 0.889 for MT segmentation; 0.977, 0.877, and 0.924 for MRC segmentation; 1, 0.942, and 0.970 for sinusitis segmentation. However, only maxillary sinus regions with clear borders were included in the segmentation area, and the data were also acquired from a single center. Hung et al.^33^ grouped healthy maxillary sinuses and maxillary sinus diseases on CBCT images acquired at different radiation doses and segmented them using support vector regression (SVR) to identify MTs and MRCs and used V-Net to segment the maxillary sinus air spaces. This two-stage model yielded AUC values of 0.91 and 0.84 for MTs and MRCs, respectively, for low-dose scans, and 0.89 and 0.93 for MTs and MRCs, respectively, for full-dose scans. However, the authors reported that the model produced a higher uncertainty on CBCT scans with previous surgery or trauma in the sinus region, and on scans with artifacts present in the sinus region.

Morgan et al.^34^ found that their 3D U-Net architecture–based CNN model for fully automatic maxillary sinus segmentation on images acquired by two CBCT devices provided faster, more exact, and consistent automatic segmentation than semiautomatic methods. However, in their study, healthy and pathological maxillary sinuses were segmented together, and the maxillary sinus lesions were not segmented separately. Lastly, Kim et al.^35^ created SinusNet, a DL-based model that performed label-free maxillary sinus segmentation on CBCT images. By placing pixel-based synthetic lesions on CBCT images of maxillary sinus regions, this model enabled the segmentation of maxillary sinus lesions without labeling. It is worth mentioning that none of these models was validated on an external dataset.

From a clinical perspective, the current model is capable of delineating the proximity of root apices to the sinus floor, which may significantly influence the planning of primary endodontic treatment and the decision-making process for managing post-treatment disease. Additionally, it segments the entire maxillary sinus and the area of mucosal thickening, thereby providing quantitative data on changes in sinus volume due to cysts and tumors and could offer guidance for sinus lift elevation during implant surgery.

The strengths of this study include the heterogeneous sourcing of a balanced dataset, the implementation of novel custom models utilizing state-of-the-art algorithms, and several techniques to mitigate overfitting, in addition to external validation.

The principal methodological limitations of this study involve the use of a multi-planar 2D segmentation approach and a limited field of view (FOV). This framework was selected to ensure computational feasibility and to capitalize on the high in-plane resolution of the available data. While this approach sacrifices the spatial context of a 3D volumetric analysis, it enabled rapid, robust training and highly accurate slice-wise inference. Similarly, the limited FOV strategically concentrated the model’s training and predictions on the dentoalveolar region of the sinus. While this excluded the superior recesses, it prioritized the anatomical features most critical for dental and implant-related planning.

The present work thus establishes a validated foundational framework. Future research can directly build upon this architecture to incorporate full-volume 3D data. This would avoid missing important volumetric patterns such as the continuity of a mucosal thickening across multiple slices or the full extension of a root infection, thereby extending the model’s applicability to a broader range of clinical and diagnostic tasks. Future research can also consider a subclassification of MS-NEO to include mucous retention cysts, polypoidal sinusitis and other sinus changes due to allergy, viral infections or upper respiratory tract infections. Also, expanding validation to additional clinical centers, scanner manufacturers, and larger patient populations would provide stronger evidence of model capability.

## Conclusion

The proposed algorithm represents a significant advancement in the domain of maxillary sinus assessment. By integrating a sequence of models to classify image views, segment maxillary sinus regions, extract radiographic features and classify sinus conditions. This approach, integrated with thorough clinical examination, offers a comprehensive and automated solution for analyzing CBCT sinus images.

## Supplementary Information

Supplementary Information 1.

Supplementary Information 2.

Supplementary Information 3.

Supplementary Information 4.

## Data Availability

All data supporting the findings of this study are available within the paper and its Supplementary Information.
