# Supplementary material for: Detection of maxillary sinusitis of endodontic origin in cone-beam CT images using deep learning algorithms
Source: Sci Rep. 2026 May 26;16:16254. doi: 10.1038/s41598-026-52147-w (PMC13212594; doi:10.1038/s41598-026-52147-w)
Supplement: Supplementary file 1 — Supplementary Information 1. [file 41598_2026_52147_MOESM1_ESM.pdf]

FD BUE REC
The British University in Egypt
Faculty of Dentistry
Research Ethics Committee

Research Report

Candidate name: Shehab El Din Mohamed Saber, Ahmed Fahmi, Nora Saif Elnasr Taha, Omar Ayman, Huda Mohammed

Degree: research paper

Department: Endodontics

Research title: Automated diagnosis of odontogenic sinusitis of endodontic origin

Status:

- ■ Initial review
- ☐ Continuing review
- ☐ Final Report

Upon reviewing the research with reference to the scientific research ethics charter, the Ethics committee decision is to.

- ☐ Approved as submitted
- ☐ Approved with minor modifications as listed below
- ☐ Postpone the decision to obtain more information as listed below
- ☐ Disapprove the protocol for the reasons listed below.
- ■ Exempt from approval of research ethic committee as it does not fall under the umbrella of human subjects and exempt from continuing review.

Date: 10/12/2024

Modifications:

Information needed:

Reasons for disapproval:

Research Approval Number: 24-075

FD BUE REC
IRB00012490
RHDIRB2020061401

Head of REC:
Prof. Dr. /Dalia Ghalwash
