# Supplementary figures and images for: Detection of maxillary sinusitis of endodontic origin in cone-beam CT images using deep learning algorithms
Source: Sci Rep. 2026 May 26;16:16254. doi: 10.1038/s41598-026-52147-w (PMC13212594; doi:10.1038/s41598-026-52147-w)

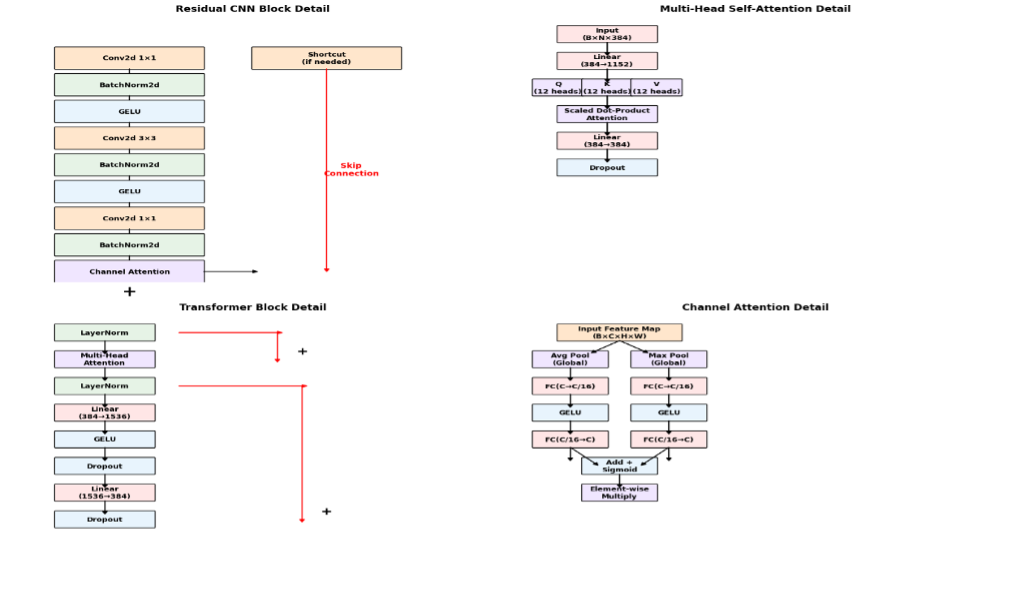

Supplement: Supplementary file 3 — Supplementary Information 3. [file 41598_2026_52147_MOESM3_ESM.png]

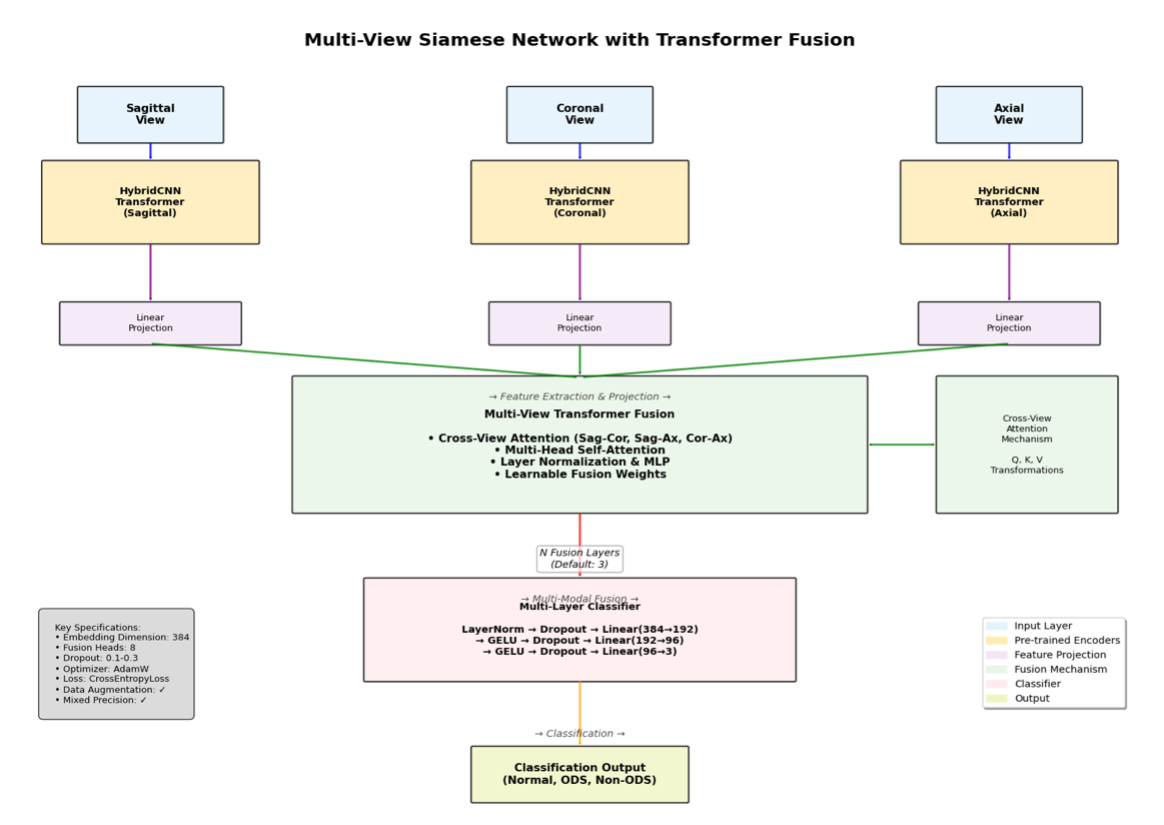

Supplement: Supplementary file 4 — Supplementary Information 4. [file 41598_2026_52147_MOESM4_ESM.png]
